# GSK-3*β*-dependent downregulation of *γ*-taxilin and *α*NAC merge to regulate ER stress responses

**DOI:** 10.1038/cddis.2015.90

**Published:** 2015-04-16

**Authors:** Y Hotokezaka, I Katayama, K van Leyen, T Nakamura

**Affiliations:** 1 Department of Radiology and Cancer Biology, Nagasaki University School of Dentistry, 1-7-1 Sakamoto, Nagasaki 852-8588, Japan; 2 Neuroprotection Research Laboratory, Departments of Radiology and Neurology, Massachusetts General Hospital and Harvard Medical School, Charlestown, MA 02129, USA

## Abstract

The signaling pathway leading to the endoplasmic reticulum (ER) stress responses has not been fully elucidated. Here we showed that glycogen synthase kinase-3*β* (GSK-3*β*)-dependent downregulation of *γ*-taxilin and nascent polypeptide-associated complex *α*-subunit (*α*NAC) mediates hypoxia-induced unfolded protein responses (UPRs) and the subsequent apoptotic and autophagic pathways. The degradation of *γ*-taxilin or *α*NAC was sufficient to initiate UPRs in normoxic cells. However, the ER stress signaling pathways initiated by *γ*-taxilin or *α*NAC were distinct, triggering different ER stress sensors and activating different downstream pathways. Hypoxia caused GSK-3*β*-dependent tau hyperphosphorylation and cleavage in neuronal cells, but *γ*-taxilin ablation induced tau hyperphosphorylation alone and *α*NAC ablation induced neither changes. Notably, downregulation of *γ*-taxilin and *α*NAC occurs in the brain of patients with Alzheimer's disease. These results suggest that GSK-3*β*-dependent downregulation of *γ*-taxilin and *α*NAC, which differently activate the UPRs, merge to regulate hypoxia-induced ER stress responses and provide a new insight into the pathogenesis of neurodegenerative diseases.

Hypoxia occurs in solid tumors and neurodegenerative diseases owing to an inadequate supply of oxygen. Prolonged hypoxic stresses may lead to the activation of unfolded protein responses (UPRs). Recent studies revealed the involvement of hypoxia-inducible factors (HIFs) and mammalian target of rapamycin (mTOR) in the initiation of responses to hypoxia.^1, 2, 3^ These independently activated signaling events (HIF, mTOR, and UPR) promote hypoxia tolerance by regulating mRNA transcription and protein translation in hypoxic cells. However, the molecular mechanisms that link between hypoxic stresses and these signaling pathways are not fully understood.

The UPR pathway, which is initiated at the endoplasmic reticulum (ER) owing to the accumulation of unfolded and misfolded proteins in the ER lumen, can be induced by hypoxia as well.^4^ To date, three ER-resident transmembrane proteins have been identified as UPR sensors: inositol-requiring protein-1*α* (IRE1*α*), protein kinase RNA-like ER kinase (PERK), and activating transcription factor 6 (ATF6). Although the sensor proteins are all activated after dissociation from the intraluminal chaperone Ig-binding protein (BiP),^5^ the signaling pathways activated by each of these sensors are unique.^6^ However, the mechanism by which unfolded and misfolded proteins accumulate in the ER lumen in hypoxic cells is not well understood.

The syntaxin-binding taxilin protein family is implicated in intracellular vesicle trafficking, and some syntaxin family members are localized on the ER and Golgi.^7^ Furthermore, *γ*-taxilin, which is ubiquitously expressed and preferentially interacts with syntaxin-4,^8^ can interact with ATF4, which controls the transcription of genes involved in ER stress-induced apoptosis.^9^

The nascent polypeptide-associated complex (NAC) is a dimeric complex of *α*NAC and *β*NAC subunits. The *α*NAC subunit can bind growing nascent chains (NCs) emerging from the ribosome and modulate the action of signal recognition particles, which transport the NCs into the ER lumen.^10, 11^ The *α*NAC has been implicated as a component of the ribosomal exit tunnel, providing a shield for NCs, and acting as a negative regulator of NC translocation into the ER. However, these functions of NAC are under debate, and some researchers favor the notion that *α*NAC serves as a transcriptional coactivator.^12^ Recently, Hotokezaka *et al.*^13^ demonstrated that ablation of *α*NAC can initiate ER stress responses and subsequent apoptosis, suggesting a role for *α*NAC in the proper translocation of NCs into the ER. However, the role of *γ*-taxilin in the UPR and subsequent apoptotic signaling pathways is still to be clarified. Another study suggests physical interaction of *γ*-taxilin and *α*NAC.^14^ Interestingly, the *β*NAC subunit was implicated as having apoptosis-suppressing activity at the mitochondria in *Caenorhabditis elegans*.^15^

These results raise the possibility that the loss of *γ*-taxilin and *α*NAC in hypoxia cooperatively causes accumulation of misfolded and unfolded proteins in the ER and initiates the UPR pathway in these cells. Here, using *in vitro* and *ex vivo* hypoxia models, we demonstrate that downregulation of *γ*-taxilin or *α*NAC mediate persistent hypoxic signals to UPRs, leading to apoptotic cell death. Further, we indicate the involvement of *γ*-taxilin in the development of brain tauopathy including Alzheimer's disease (AD).

## Results

### *γ*-Taxilin downregulation in hypoxic cells

**Figure 1:**
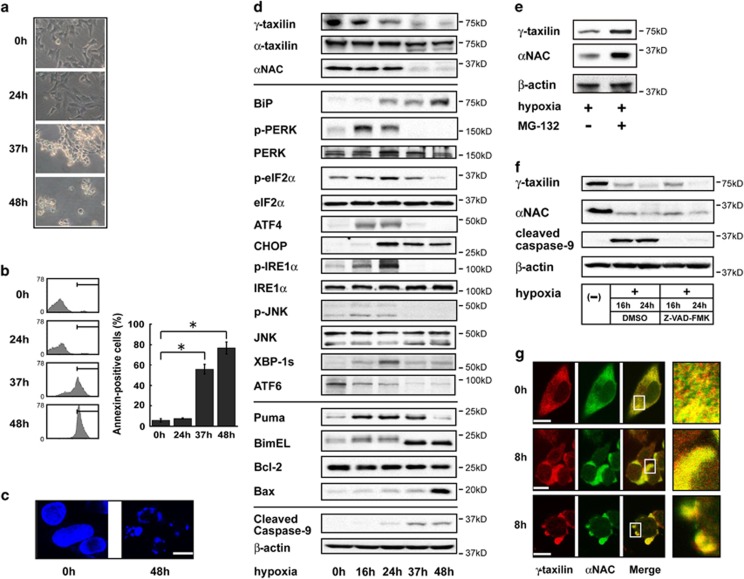
Downregulation of *γ*-taxilin and *α*NAC and ER stress responses in hypoxic cells. (**a**) Phase-contrast micrographs of SK-N-SH cells cultures in hypoxic conditions for 0–48 h. Floating cells were evident by 37 h after the beginning of hypoxic treatment. (**b**) FACS (fluorescence-activated cell sorting) analysis of apoptosis in hypoxic SK-N-SH cells. Bars in the FACS profiles (left panel) indicate the fraction locations of annexin-positive cells. Bar graph indicates the relative numbers of annexin-positive cells (right panel). Bar graph data are shown as means±S.D. (*n*=3). *Significantly different from controls (0 h) (*P*<0.001, Tukey–Kramer test). (**c**) DAPI (4',6-diamidino-2-phenylindole) staining shows fragmented nuclei of apoptotic SK-N-SH cells after hypoxic treatment. Scale bar, 10 *μ*m. (**d**) Downregulation of *γ*-taxilin and *α*NAC and induction of ER stress responses in hypoxic SK-N-SH cells. Western blot analysis was performed 0 to 48 h after hypoxic treatment of cells. Upper panels, taxilin and NAC proteins; middle panels, UPR proteins; lower panels, apoptotic proteins; and bottom panels, cleaved caspase-9 and *β*-actin proteins. (**e**) MG-132 treatment restore the *γ*-taxilin and *α*NAC protein level in hypoxic (16 h) HeLa S3 cells. MG-132 (−), DMSO; MG-132 (+), 20 *μ*M. (**f**) Caspase inhibition does not affect the hypoxia-induced downregulation of *γ*-taxilin and *α*NAC in HeLa S3 cells. (**g**) Colocalization of *γ*-taxilin and *α*NAC in normoxic (0 h) and hypoxic (8 h) HeLa S3 cells. Note that the colocalization fraction increases with the appearance of apoptotic phenotypes. Scale bars, 10 *μ*m

In response to prolonged (>16 h) hypoxia, *γ*-taxilin protein levels were downregulated in SK-N-SH human neuroblastoma cells (Figures 1a–d). Along with these events, the hypoxic cells initiate the UPR and apoptotic pathways by activating ER stress sensor proteins such as PERK and IRE1*α* and their downstream targets (Figure 1d). However, the levels of the third ER stress sensor protein, ATF6, were decreased and not cleaved in hypoxic cells (Figure 1d). The taxilin family is composed of at least three members, *α*, *β*, and *γ*.^8^ We found that although *α*-taxilin was expressed in SK-N-SH cells, the protein levels did not significantly change after induction of hypoxia (Figure 1d). On the other hand, *β*-taxilin was not detected in these cells. These results suggest that hypoxia specifically downregulates *γ*-taxilin. MG-132 treatment restored the *γ*-taxilin and *α*NAC protein levels in hypoxic HeLa S3 cells, suggesting that the downregulation occurred through posttranslational modifications (Figure 1e). A pancaspase inhibitor, Z-VAD-FMK, did not affect *γ*-taxilin or *α*NAC protein levels, indicating that the hypoxia-induced degradation of these proteins is caspase-independent (Figure 1f).

### Colocalization of *γ*-taxilin and *α*NAC in normoxic and hypoxic cells

Physical interaction between *γ*-taxilin and *α*NAC was suggested to occur in COS-7 cells overexpressing *γ*-taxilin and *α*NAC.^14^ Therefore, we investigated the intracellular distribution of these two proteins in normoxic and hypoxic cells by using confocal microscopy. In normoxic cells, *γ*-taxilin and *α*NAC were distributed throughout the cytoplasm and a large proportion of both proteins was colocalized (Figure 1g). In hypoxic cells, most of the *γ*-taxilin and *α*NAC proteins were still colocalized throughout the cytoplasm and in the hypoxic blebs.

### *γ*-Taxilin degradation in hypoxic brain slice cultures

**Figure 2:**
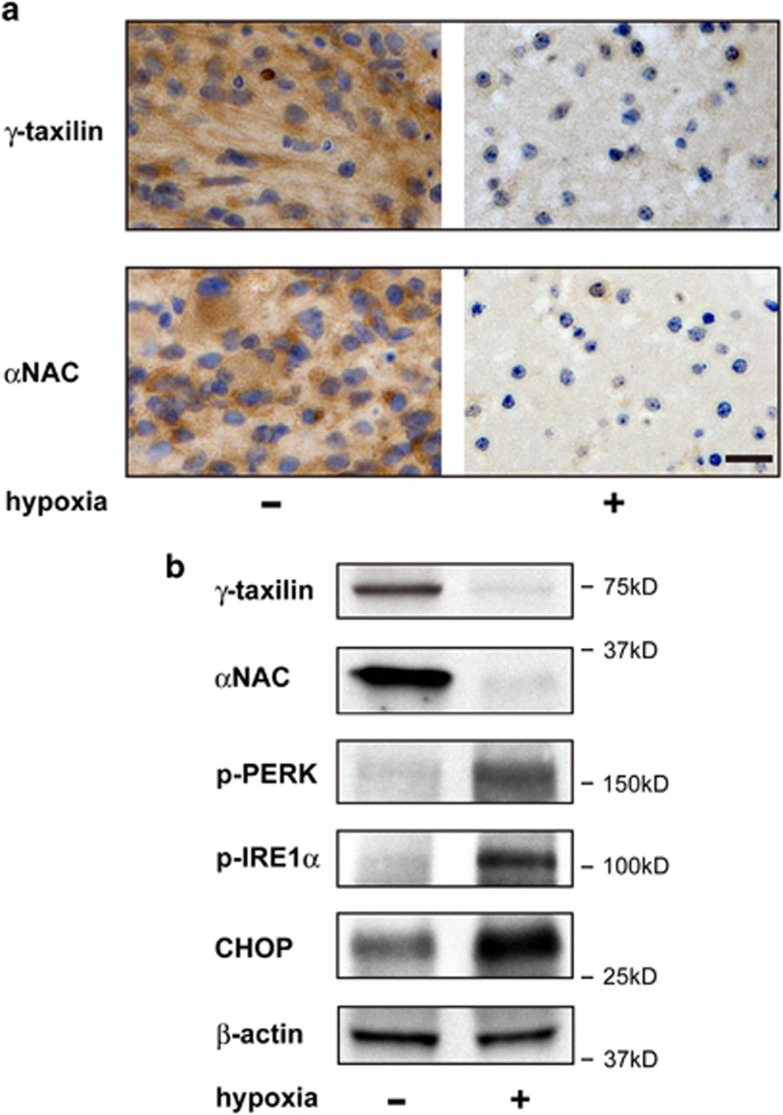
Downregulation of *γ*-taxilin and *α*NAC, and ER stress responses in mouse brain slice cultured under hypoxic conditions. (**a**) Immunohistochemistry shows downregulation of *γ*-taxilin and *α*NAC expression in brain slices cultured for 48 h under hypoxic conditions. Scale bar, 20 *μ*m. (**b**) Western blot shows downregulation of *γ*-taxilin and *α*NAC, phosphorylation of PERK and IRE1*α* and induction of CHOP in mouse brain slices cultured for 48 h under normoxic (−) or hypoxic conditions (+)

To further extend these findings, we tested whether similar events could occur in *ex vivo* conditions. To this end, we used mouse brain slices.^16^ Hypoxia-induced downregulation of *γ*-taxilin was confirmed in hypoxic brain slice cultures (Figure 2). We also confirmed that downstream ER stress response pathways were activated as in the culture systems. *α*NAC was also downregulated in the hypoxic brains as described previously.^13^

### UPRs initiated by *γ*-taxilin depletion

**Figure 3:**
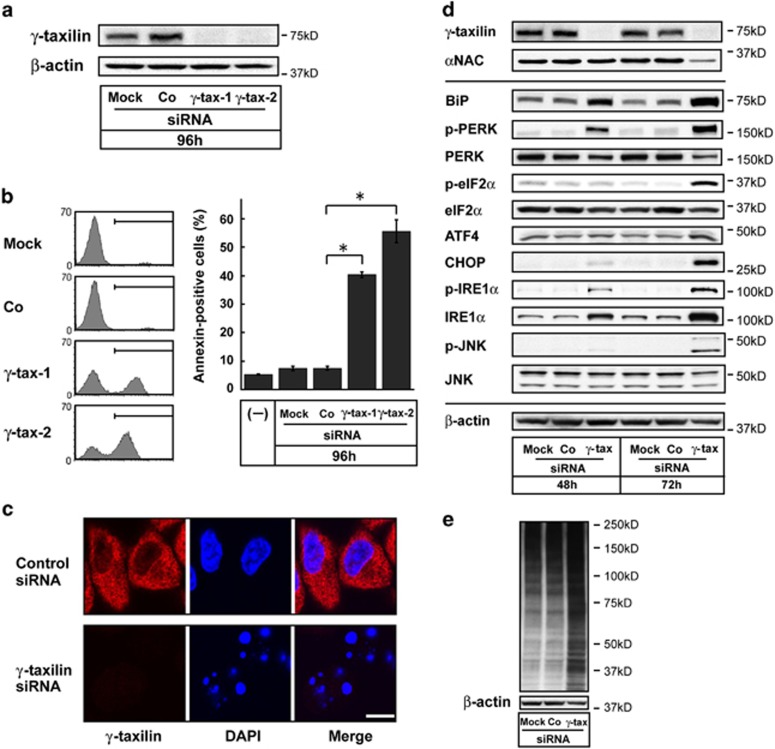
*γ*-Taxilin ablation causes ER stress responses and apoptosis in normoxic cells. (**a**) Knockdown of *γ*-taxilin expression by small interfering RNA (siRNA) in HeLa S3 cells. Two different siRNA constructs are equally effective. HeLa S3 cells were treated with solvent alone (Mock), control siRNA (Co), or *γ*-taxilin siRNA (*γ*-tax-1 or -2) for 96 h. (**b**) Induction of apoptosis by *γ*-taxilin ablation in HeLa S3 cells. *γ*-Taxilin ablation causes apoptosis in HeLa S3 cells, but Mock or Co treatment did not. Bar graph shows fractions of annexin-positive cells (means±S.D., *n*=3). *Significantly different from Mock- or Co treatment (*P* <0.001, Tukey–Kramer test). (**c**) Confocal microscopy demonstrates coincidence of *γ*-taxilin depletion and apoptotic nuclei in HeLa S3 cells treated with *γ*-taxilin siRNA. Scale bar, 10 *μ*m. (**d**) *γ*-Taxilin depletion triggers ER stress responses in HeLa S3 cells. Upper panels, taxilin and NAC proteins; middle panels, UPR proteins; and lower panel, *β*-actin. (**e**) *γ*-Taxilin ablation induces accumulation of ubiquitinated proteins in HeLa S3 cells. Cell lysates were analyzed on 7.5% SDS-PAGE, followed by immunoblotting with antibodies specific for anti-mono- and polyubiquitinated conjugates (upper panel) and *β*-actin (lower panel)

Given the comparable degradation of *γ*-taxilin in hypoxic cells, we next tested whether *γ*-taxilin depletion alone could invoke ER stress responses in the absence of hypoxia. We found that *γ*-taxilin ablation, effectively achieved by using *γ*-taxilin-specific siRNAs (*γ*-tax-1 or *γ*-tax-2), resulted in apoptotic cell death (Figures 3a–c).

ER stress signaling pathways involve three distinct stress sensor proteins, IRE1, PERK, and ATF6.^17^ BiP is a negative regulator of these sensor proteins.^18^ Since unfolded proteins that accumulate in the ER can bind BiP and sequester it from the sensor molecules, we first monitored the expression levels of BiP in *γ*-taxilin-depleted HeLa S3 cells. We found that BiP levels were elevated 48 h after the addition of *γ*-taxilin siRNA and continued to increase up to at least 72 h after siRNA addition (Figure 3d).

The dissociation of BiP from PERK leads to autophosphorylation of PERK, which in return phosphorylates eIF2*α*. The phosphorylation of eIF2*α* reduces the formation of translation initiation complexes and thus reduces the general rate of translation initiation.^6^ Therefore, we tested whether similar events occur in *γ*-taxilin-depleted cells. We found that *γ*-taxilin ablation by the RNA interference activated PERK and elF2*α* in HeLa S3 cells (Figure 3d).

Translation of ATF4 mRNA requires phosphorylation of eIF2*α*.^19^ The ATF4 protein then directly binds to the C/EBP homologous protein (CHOP) promoter and CHOP protein synthesis is induced.^20^ Consistent with this model, phosphorylated eIF2*α* and ATF4 protein levels increased, along with the upregulation of CHOP protein levels in *γ*-taxilin-depleted cells (Figure 3d). Cleavage of ATF6, which is rapidly initiated after exposure to ER stress,^6^ can upregulate CHOP protein levels.^20^ However, the level and size of the ATF6 protein were not affected by the *γ*-taxilin ablation as in the hypoxic cells (Supplementary Figure S1). Therefore, the upregulation of CHOP protein by the *γ*-taxilin ablation may be caused by the PERK/eIF2*α*/ATF4 signaling pathway.

The release of BiP allows IRE1*α* to dimerize and autophosphorylate, removing a 26-base intron from X-box-binding protein-1 (XBP-1) mRNA.^21^ The spliced XBP-1 then activates the expression of UPR target genes. On the other hand, the dimerized IRE1*α* can activate c-Jun N-terminal kinase (JNK) after complex formation with tumor necrosis factor-*α*-receptor-associated factor 2.^22, 23^ A third sensor, ATF6, induces XBP-1 mRNA, which is then spliced by IRE1*α* in response to ER stress.^24, 25^ As expected from the absence of ATF6 cleavage, spliced XBP-1 levels did not change after *γ*-taxilin ablation (Supplementary Figure S1). However, we found that *γ*-taxilin ablation results in the phosphorylation of IRE1*α* protein and activation of JNK (Figure 3d). These results suggest that the *γ*-taxilin depletion-induced UPRs occurred chiefly, if not exclusively, through the PERK-elF2*α* and IRE1*α*-JNK sensor pathways.

### Ubiquitin accumulation in *γ*-taxilin-depleted cells

Unfolded or misfolded proteins that are not transported from the ER to the Golgi compartment are degraded via the ubiquitin/proteasome pathway.^26^ Previously, we found that knockdown of *α*NAC enhanced protein ubiquitination.^13^ Therefore, we surmised that ubiquitin must accumulate in the cytoplasm to degrade the unfolded or misfolded proteins in *γ*-taxilin-depleted cells (Supplementary Figure S2). To test this possibility, we performed western blot analysis using an antibody specific for ubiquitin.^27^ The western blot analysis demonstrated increases in ubiquitinated proteins in the *γ*-taxilin-depleted cells, but not in mock-treated or control cells (Figure 3e).

### Mitochondria-dependent apoptotic pathway in *γ*-taxilin-depleted cells

**Figure 4:**
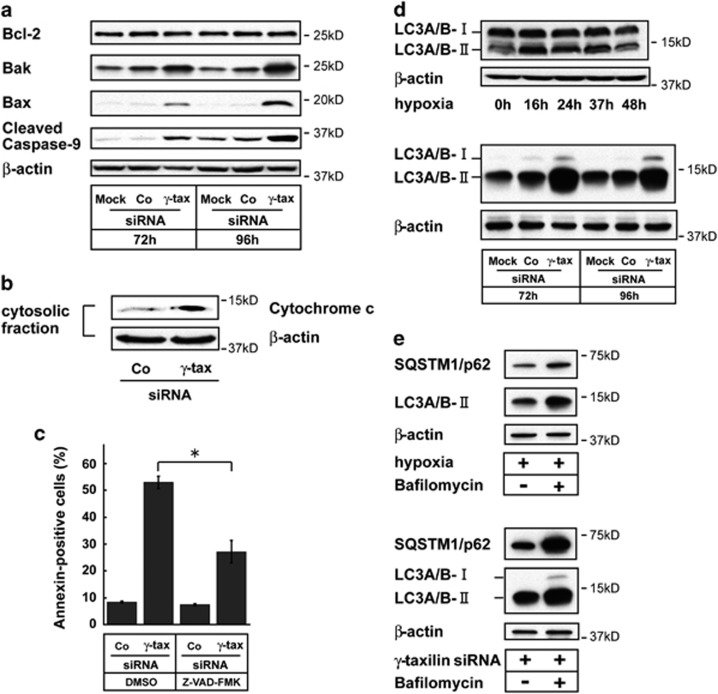
*γ*-Taxilin ablation-induced apoptosis is mitochondria- and caspase-dependent. (**a**) *γ*-Taxilin ablation of HeLa S3 cells activate Bak, Bax, and caspase-9 activity, but did not affect Bcl-2. (**b**) Enhanced release of cytochrome *c* in *γ*-taxilin-depleted HeLa S3 cells. (**c**) Suppression of apoptosis by caspase inhibition in *γ*-taxilin-depleted cells. Bar graph shows fractions of annexin-positive HeLa S3 cells treated with control (Co) or *γ*-taxilin small interfering RNA (siRNA) (*γ*-tax) in the presence or absence of a pancaspase inhibitor Z-VAD-FMK (100 *μ*M). *Significantly different (*P*<0.001, Tukey–Kramer test). (**d**) LC3-II (LC3A/B-II) upregulation in hypoxic SK-N-SH cells (upper panel) and in *γ*-taxilin-depleted HeLa S3 cells (lower panel). LC3A/B-I (16 kDa) and LC3A/B-II (14 kDa). (**e**) Bafilomycin upregulates SQSTM1/p62 and LC3A/B-II protein levels in hypoxic and *γ*-taxilin-depleted HeLa S3 cells. Cells were cultivated in hypoxic conditions for 24 h in the presence of bafilomycin (200 nM, upper panel) or were treated with *γ*-taxilin for 96 h in the presence of bafilomycin for the last 24 h

One of the widely cited mechanisms of CHOP-induced apoptosis is suppression of the prosurvival protein Bcl-2.^28^ We found that the Bcl-2 protein levels were not affected in *γ*-taxilin-depleted cells (Figure 4a). *γ*-Taxilin ablation was associated with Bax and Bak protein upregulation, which is thought to bind the mitochondrial outermembrane and thereby causing cytochrome c release. All pathways to apoptosis converge on the activation of caspases.^29^ The mitochondrial apoptotic pathway involves the activation of caspase-9.^30^ Consistent with those notions, we confirmed that *γ*-taxilin ablation-induced cell death is caspase-9-dependent, but not on caspase-4 (Figures 4a–c; Supplementary Figure S1). These results suggest that the *γ*-taxilin ablation-induced apoptosis is Bcl-2-independent and mitochondria-dependent.

### Autophagy in *γ*-taxilin-knockdown cells

Next, we tested the possibility of autophagy involvement in cell death provoked by *γ*-taxilin depletion. To this end, we performed western blot analysis using anti-LC3-II antibodies. We found that autophagy contributed at least in part to the cell death caused by *γ*-taxilin knockdown or hypoxia (Figure 4d). A lysosomal inhibitor bafilomycin blocks autophagosome–lysosome fusion.^31^ Therefore, the inhibitor would increase the intracellular protein levels of SQSTM1/p62 as well as LC3-II in cells undergoing autophagic cell death. As expected, bafilomycin upregulated these protein levels in hypoxic and *γ*-taxilin-depleted cells (Figure 4e).

### Differential ER stress responses to *γ*-taxilin and *α*NAC depletion

**Figure 5:**
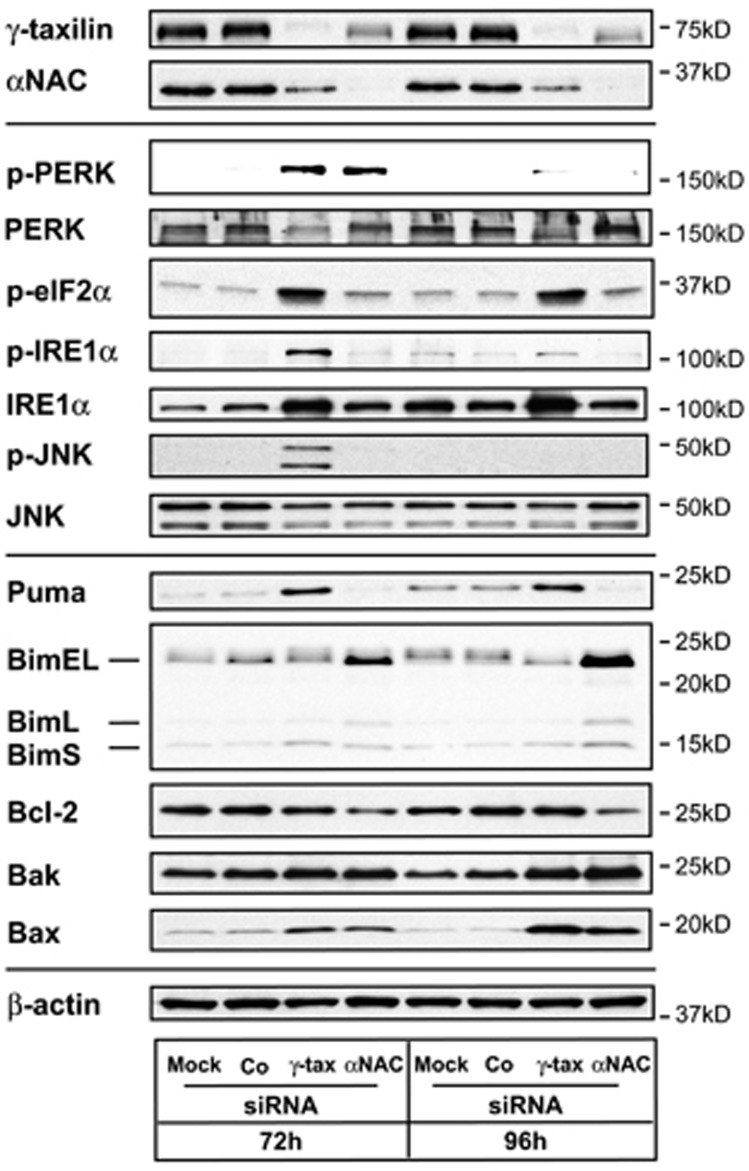
Differential ER stress response pathways initiated by *γ*-taxilin or *α*NAC depletion. Proteins involved in UPR sensor (PERK, IRE1*α*), eIF2*α*, JNK, Bcl-2, and Bcl-2-related protein (Puma, Bim, Bak, and Bax) signaling pathways were analyzed by Western blotting of HeLa S3 cells that were depleted of *γ*-taxilin or *α*NAC by RNA intereference for 72 and 96 h. Upper panels, taxilin and NAC proteins; middle panels, UPR proteins; lower panels; apoptotic proteins; and bottom panel, *β*-actin

The ablation of *γ*-taxilin was associated with large decreases in *α*NAC protein levels, and *α*NAC silencing was associated with large decreases in the amount of *γ*-taxilin protein (Figure 5). These results imply that the presence of *α*NAC is required for maintaining the *in vivo* protein level of *γ*-taxilin, and *vice versa*. Therefore, we compared the expression profiles of proteins involved in the UPR and apoptosis pathways between cells treated with *γ*-taxilin and those treated with *α*NAC siRNA.

Distinctive profiles of ER stress responses between the *γ*-taxilin-knockdown cells and *α*NAC-knockdown cells were found for IRE1*α*, JNK, and Puma (p53- upregulated modulator of apoptosis) (Figure 5). These proteins were activated in *γ*-taxilin-knockdown cells, but not in *α*NAC-knockdown cells. On the other hand, Bim upregulation and Bcl-2 downregulation occurred in *α*NAC-knockdown cells, but not in *γ*-taxilin-knockdown cells. Therefore, these results suggest that the roles of *γ*-taxilin and *α*NAC in ER stress responses are divergent.

Overexpression of *α*NAC partially rescues hypoxic cells from apoptosis.^13^ Therefore, we tested the possibility that *γ*-taxilin overexpression could also rescue hypoxic cells. However, we found that overexpression of *γ*-taxilin accelerated apoptosis of normoxic cells (Supplementary Figure S3a). We also investigated whether the overexpression of *α*NAC could alleviate the apoptotic cell death induced by *γ*-taxilin ablation. Results indicated that *α*NAC overexpression did not affect the rate of *γ*-taxilin siRNA-induced cell death (Supplementary Figure S3b). Collectively, these results support the notion that *γ*-taxilin and *α*NAC depletion invoke different mechanisms of ER stress responses in the cell.

### GSK-3*β*-dependent degradation of *γ*-taxilin in hypoxic cells

**Figure 6:**
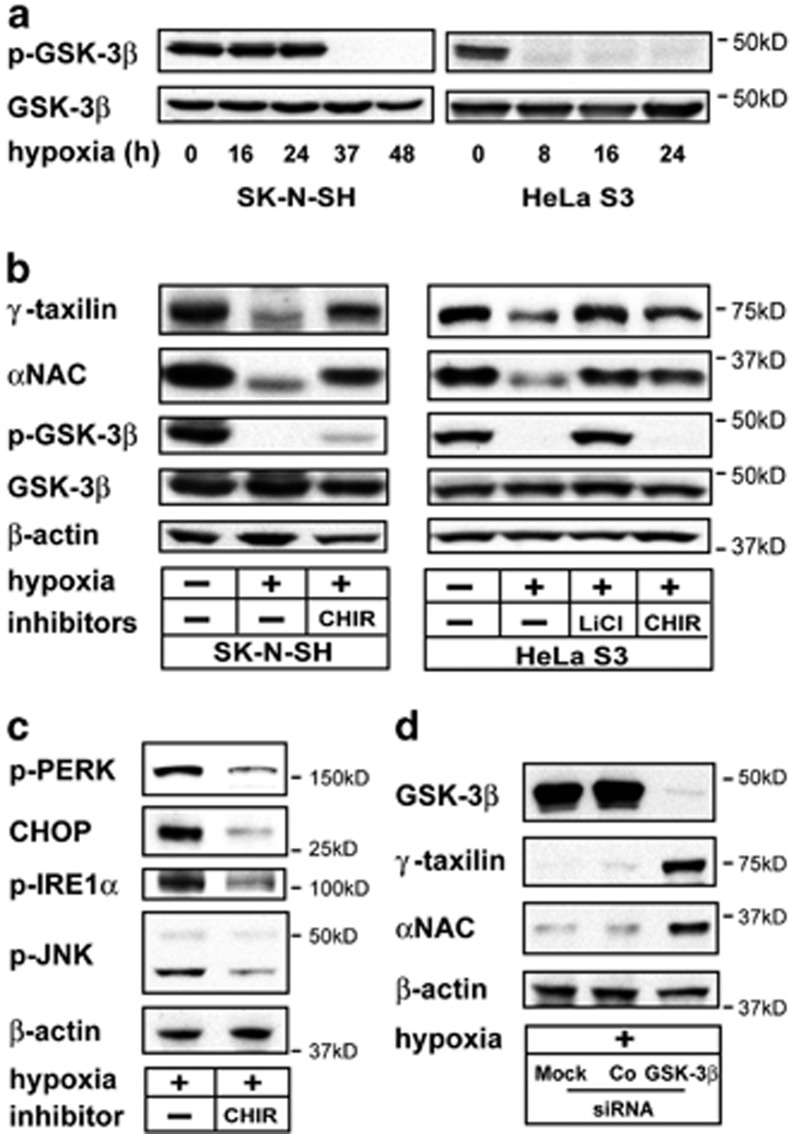
Hypoxia-induced downregulation of *γ*-taxilin and *α*NAC and the subsequent ER responses are GSK-3*β*-dependent. (**a**) GSK-3*β* activation in hypoxia. Western blot analysis shows downregulation of phosphorylated (Ser 9) GSK-3*β* (phospho-GSK-3*β*) in hypoxic (0–48 h) SK-N-SH and hypoxic (0–24 h) HeLa S3 cells. (**b**) Inhibition of GSK-3*β* activation by lithium chloride (LiCl, 100 mM) or CHIR-99021 (CHIR, 15 or 30 *μ*M) maintained *γ*-taxilin and *α*NAC protein levels in hypoxic SK-N-SH and HeLa S3 cells. (**c**) GSK-3*β* inhibition suppresses the expression of ER stress response proteins that are induced in hypoxic SK-N-SH cells. (**d**) GSK-3*β* RNA interference almost completely blocked the protein expression and restored the expression of *γ*-taxilin and *α*NAC proteins in hypoxic HeLa S3 cells

We found that most, if not all, of the glycogen synthase kinase-3*β* (GSK-3*β*) proteins were dephosphorylated at Ser 9 in cells cultured in hypoxic conditions (Figure 6a). Considering the GSK-3*β*-dependent *α*NAC degradation^13, 32^ and GSK-3*β*-dependent apoptosis in hypoxia,^33^ we expected that GSK-3*β* inhibition could restore *γ*-taxilin as well as *α*NAC protein levels in hypoxic cells. As expected, GSK-3*β*-specific inhibitors restored *γ*-taxilin as well as *α*NAC protein levels and suppressed hypoxia-induced cell death (Figure 6b; Supplementary Figure S4).^34, 35^ The CHIR-99021 also substantially suppressed the hypoxia-induced activation of PERK, CHOP, IRE1*α*, and JNK, a finding that was consistent with the rescue of apoptosis by GSK-3*β* inhibition (Figure 6c).

More specifically, we performed GSK-3*β* RNA interference to confirm the GSK-3*β* dependency of *γ*-taxilin downregulation in hypoxia. We found that GSK-3*β*-specific siRNA, which almost completely inhibited the protein expression, restored *γ*-taxilin and *α*NAC protein levels of hypoxic cells (Figure 6d).

To explore the possible GSK-3*β* phosphorylation site(s) of *γ*-taxilin in hypoxic cells, we conducted mass spectrometry using hypoxic HeLa S3 cells. The mass spectrometry revealed that *α*NAC was thereonine phosphorylated at ^157^TQTPT^161^, which was the reported consensus phosphorylation site for GSK-3*β*.^32^ However, *γ*-taxilin was not phosphorylated in these cells, implying that *γ*-taxilin was degraded by a distinct mechanism from *α*NAC, which might be mediated by an undefined substrate(s) of GSK-3*β*.

### Tau hyperphosphorylation in hypoxic and *γ*-taxilin-depleted cells

**Figure 7:**
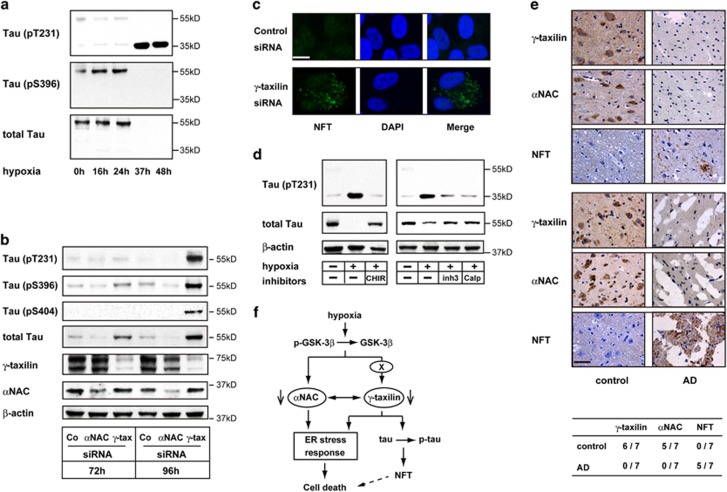
Hypoxia or *γ*-taxilin ablation induces tau hyperphosphorylation and cleavage in neuronal cells. (**a**) Western blot shows hyperphosphorylation at T231 (upper panel) and S396 (middle panel), and tau protein (~55 kDa) (lower panel) in SK-N-SH neuronal cells cultured under hypoxic conditions for indicated durations. Note that cleaved (~35 kDa) tau contains phosphorylated T231, but not phosphorylated S396. (**b**) Western blot shows hyperphosphorylation of tau protein (T231, S396, and S404) in SH-SY5Y neuronal cells cultured in the presence of *γ*-taxilin-specific small interfering RNA (siRNA), but not in *α*NAC siRNA-treated cells. Note that total tau increases in amount after *γ*-taxilin-specific siRNA addtion. (**c**) Confocal microscopy shows tau aggregations (NFT) in the cytoplasm of *γ*-taxilin-specific siRNA-treated SH-SY5Y cells. Scale bar, 10 *μ*m. (**d**) Tau cleavage is calpain-dependent. Western blot analysis shows that calpain inhibitors (inh3, calpain inhibitor III; or calp, calpeptin) inhibit tau cleavage, but not tau hyperphosphorylation in SK-N-SH neuronal cells (right panels). As expected, GSK-3*β* inhibition with CHIR inhibits tau hyperphosphorylation in the neuronal cells. (**e**) Downregulation of *γ*-taxilin and *α*NAC in the brains of patients with AD. Immunohistochemistry shows *γ*-taxilin and *α*NAC protein expression in the healthy brain (control, left panels), but not in the brains from patients with AD (right panels). Upper panels, temporal lobe; lower panels, precentral gyrus. Table shows detection rates of *γ*-taxilin, *α*NAC, and NFT in the control (*n*=7) and AD (*n*=7) brain sections. Scale bar, 200 *μ*m. (**f**) Schema shows *γ*-taxilin and *α*NAC involvement in hypoxia-induced ER stress responses and tau hyperphosphorylation pathways. *γ*-Taxilin is downstream of GSK-3*β* and upstream of tau phosphorylation, but not upstream of GSK-3*β*-dependent tau phosphorylation (Figures 6b, 7d; Supplementary Figure S5). *γ*-Taxilin degradation in hypoxia may be mediated by an undefined substrate (X) of GSK-3*β*

The microtubule-associated protein tau is the core component of neurofibrillary tangles (NFTs). Tau protein is hyperphosphorylated in response to hypoxia and is a target protein of GSK-3*β*.^36^ Therefore, we tested whether tau protein is involved in ER stress responses induced by hypoxia or in cells that are depleted of *γ*-taxilin. We found that tau protein (~55 kDa) was cleaved to ~35 kDa fragments and was hyperphosphorylated at T231 after cleavage and hyperphosphorylated at S396 before cleavage in SK-N-SH neuronal cells that were cultured under hypoxic conditions (Figure 7a). Therefore, we further tested whether *γ*-taxilin ablation can induce tau hyperphosphorylation. In SH-SY5Y neuronal cells, *γ*-taxilin ablation caused tau hyperphosphorylation at T231, S396, and S404, but protein cleavage did not occur (Figure 7b). In contrast, tau protein was not hyperphosphorylated or cleaved in *α*NAC-depleted cells. We further confirmed that NFT deposition occurred in the cytoplasm of *γ*-taxilin-depleted neuronal cells (Figure 7c).

Next, we tested whether tau hyperphosphorylation depends on GSK-3*β* activation. We found that an addition of CHIR to the culture medium inhibited the phosphorylation of tau protein in hypoxic SK-N-SH cells (Figure 7d). However, the GSK-3*β* inhibition did not completely prevent tau cleavage in the hypoxic cells. These results suggest that tau phosphorylation in hypoxia occur through a GSK-3*β*-*γ*-taxilin signaling pathway. On the other hand, the tau cleavage in hypoxic neuronal cells is attributable for the action of calpain (Figure 7d).^37, 38, 39^

### *γ*-Taxilin and *α*NAC involvement in the pathogenesis of AD

Lastly, we tested the possibility that the downregulation of *γ*-taxilin and *α*NAC might occur in the brain of patients with AD. To this end, we performed immunohistochemical analysis of *γ*-taxilin and *α*NAC using brain tissues obtained from patients with AD. We found that *γ*-taxilin and *α*NAC proteins were detected in six and five out of seven sections obtained from different parts of the control brain, respectively (Figure 7e). However, these proteins were not detected in the brain sections from seven AD patients. We confirmed that NFT was deposited in five of the AD brain sections, but not in the controls. Collectively, these results suggest that *γ*-taxilin and *α*NAC are deeply involved in the pathogenesis of neurodegenerative diseases including AD.

## Discussion

The depletion of *γ*-taxilin was associated with phosphorylation of PERK. The activated PERK phosphorylates eIF2*α*. Phosphorylation of eIF2*α* then reduces the formation of translation initiation complexes, resulting in reduced recognition of AUG initiation codons, thereby decreasing the levels of unfolded protein in the ER. On the other hand, the translation of ATF4 is enhanced by activated eIF2*α*.^40^ *γ*-Taxilin interacts with ATF4 to inhibit ATF4-mediated transcription.^9, 41^ Therefore, the ATF4 target gene CHOP can be expected to be transcriptionally activated in *γ*-taxilin-depleted cells. However, we found that CHOP levels increased before upregulation of ATF4 protein in *γ*-taxilin depletion, suggesting CHOP induction by an as-yet undefined pathway, such as mTOR.^3^

The depletion of *γ*-taxilin activated a second branch of the ER stress sensor, IRE1*α*. Lin *et al.*^23^ proposed the concept that rapid (<8 h) attenuation of IRE1*α* signaling after ER stress serves as an important factor in determining the cell death fate, based on the observation of prolonged cell survival in cells that express artificially long-lasting IRE1*α* protein. In the present study, however, the levels of phosphorylated and unphosphorylated IRE1*α* protein remained elevated for prolonged periods of *γ*-taxilin depletion and hypoxia, probably because of the sustained activation of Bak and Bax, which then form a protein complex with IRE1*α*, activating IRE1*α* signaling.^42^

The UPR and apoptotic signaling pathways display differential profiles between *γ*-taxilin- and *α*NAC-specific siRNA-treated cells: (a) IRE1*α* signaling is activated in *γ*-taxilin-depleted cells, but not in *α*NAC-depleted cells. (b) *α*NAC silencing decreases Bcl-2 protein levels but *γ*-taxilin silencing did not affect Bcl-2 protein levels. (c) *γ*-Taxilin depletion leads to the activation of the BH3 (Bcl-2 homology domain)-only protein Puma, but not the activation of Bim, whereas *α*NAC depletion leads to the activation of Bim, but not the activation of Puma. (d) JNK was phosphorylated in *γ*-taxilin-depleted cells, but not in *α*NAC-depleted cells. Bim and Puma are key regulators of ER stress-induced apoptosis.^43^ Bcl-2 counteracts the effects of BH3-only proteins and its persistent localization in the ER membrane can block apoptosis. On the other hand, proapoptotic CHOP suppresses the transcription of Bcl-2.^6^ Furthermore, IRE1*α* can physically interact with Bim and Puma proteins, participating in XBP-1 mRNA splicing,^44^ which is strictly inhibited in cells depleted of either *γ*-taxilin or *α*NAC. XBP-1 mRNA splicing and the resultant increase in activated XBP-1 cellular levels are essential for survival under conditions of prolonged ER stress.^45^ In addition, the activation of IRE1*α* in *γ*-taxilin-depleted cells activates JNK, facilitating autophagy and apoptosis. Collectively, these results suggest that XBP-1 splicing is inhibited by an undefined mechanism in cells depleted of *γ*-taxilin, where the cells are committed to UPR and subsequent apoptotic pathways; these features were not observed in hypoxic cells with activated IRE1*α*, XBP-1 splicing, and JNK nor in *α*NAC-knockdown cells without noticeable activation of IRE1*α*, XBP-1 splicing, or JNK.

It is interesting to note that *γ*-taxilin causes decreases of *α*NAC after prolonged time and the same is true for knockdown of *α*NAC in HeLa S3 cells. However, the relationship did not exist for *γ*-taxilin or *α*NAC-knockdown SH-SY5Y cells. At present, it is not clear why the mutual dependency between *γ*-taxilin and *α*NAC was not observed in the neuronal cells. One possible explanation is that the discrepancy may be attributable to the difference in cell type.

In the present study, we convincingly showed that cell death caused by *γ*-taxilin depletion is attributable to apoptosis. However, our preliminary findings that LC3-II was upregulated in the *γ*-taxilin knockdown or hypoxic cells imply the possible involvement of autophagy in these cells. It is an intriguing idea that autophagy is activated by *γ*-taxilin depletion, as ER stress is considered to be a major stimulator of autophagy.^46^ In addition, autophagy is activated in several neurodegenerative diseases, including AD,^47^ and we found that *γ*-taxilin was depleted in the AD brains. Accumulating evidences indicate that autophagy and apoptosis share common pathways. Therefore, autophagy occurring in *γ*-taxilin-depleted or hypoxic cells may allow the cells to adapt to ER stresses.^46^

It should also be noted that total IRE1*α* levels were increased by knockdown of *γ*-taxilin, but not under other conditions tested (hypoxia or *α*NAC knockdown). We speculate that upregulated IRE1*α* might be required for the full achievement of cell death in *γ*-taxilin-depleted cells, as Bim upregulation and Bcl-2 downregulation, both are considered to contribute to apoptotic cell death, were observed in *α*NAC-knockdown cells, but not in *γ*-taxilin-knockdown cells. In this regard, Wang *et al.*^48^ showed that overexpression of IRE1*α* in HEL293T cells provoked apoptotic cell death.

Inhibition of GSK-3*β* activity effectively restores the *γ*-taxilin and *α*NAC protein levels and rescues hypoxic cells from apoptosis, suggesting that hypoxia-induced apoptosis is mediated at least in part by GSK-3*β*-dependent *γ*-taxilin and *α*NAC degradation. The exact mechanism of *γ*-taxilin and *α*NAC degradation by GSK-3*β* is not clear at present. GSK-3*β* has been implicated in the phosphorylation of many proteins, including *α*NAC.^36^ However, mass spectrometric analysis did not support the notion that GSK-3*β*-dependent phosphorylation contributes the *γ*-taxilin degradation in hypoxic cells. These results imply that degradation of *γ*-taxilin in hypoxic cells and brains might be mediated by an undefined substrate of GSK-3*β* (Figure 7f).

A causal relationship between hyperphosphorylated tau and cell death is open to debate. A postulated concept about tauopathy is that the disease phenotypes are caused by loss of tau function due to hyperphosphorylation and subsequent tangle formation.^49^ However, recent studies have shown that loss of tau function is an unlikely cause of neurodegeneration.^50, 51, 52^ Morris *et al.*^36^ have proposed a tempting concept concerning hyperphosphorylated tau; they speculated that the tau protein in AD is hyperphosphorylated and released from its binding partner to counteract neuronal dysfunction in neurodegenerative diseases such as AD. With time, however, toxic tau accumulation in the cell is detrimental to cell longevity. In fact, de Calignon *et al.*^53^ convincingly showed that tangle-bearing neurons are rarely harmed and long-lived. Therefore, tangle formation is a marker of neurodegenerative processes and tangle-bearing neurons may represent survivors of the hypoxic stress that is usually associated with apoptotic death.

## Materials and Methods

### Cell culture and hypoxia treatment

HeLa S3 human cervical cancer cells were cultured in DMEM supplemented with 10% fetal bovine serum (FBS). SH-SY5Y and SK-N-SH human neuroblastoma cells were both grown in *α*-MEM supplemented with 10% FBS. The cells were cultured under hypoxic conditions (<1% *p*O_2_ and 5% *p*CO_2_) using an anaerobic culture kit (Mitsubishi Gas Chemicals, Tokyo, Japan). The system provides a hypoxic condition without affecting the pH of the medium.

### RNA interference

Oligonucleotides corresponding to human *α*NAC (5′-CCAGUCAGUAAAGCAAAACTT-3′), *γ*-taxilin (siRNA-1, 5′-GCAAGAAUCAAGAGAGGAATT-3′; siRNA-2, 5′-GCAGAGAACUUCAGCGUCATT-3′), and GSK-3*β* (5′-CCACAAGAAGUCAGCUAUATT-3′) were transfected into HeLa S3 and SH-SY5Y cells using Lipofectamine RNAiMax (Invitrogen, Carlsbad, CA, USA) according to the manufacturer's instruction. The effect of siRNA was measured 48–96 h after the transfection. AllStars Negative Control siRNA (Qiagen, Hilden, Germany) was used as a control.

### Proteosome, GSK-3*β*, caspase, and calpain inhibition

A proteasome inhibitor MG-132 (20 *μ*M; Calbiochem, San Diego, CA, USA) was added to the hypoxic culture of HeLa S3 cells. Lithium chloride (Sigma, St. Louis, MO, USA) and CHIR-99021 (Stemgent, Cambridge, MA, USA) were used as pharmacological inhibitors of GSK-3*β*. Z-VAD-FMK (BD Pharmingen, Franklin Lakes, NJ, USA) was used as a pancaspase inhibitor. Calpain inhibitor III or calpeptin (Calbiochem) were used as calpain inhibitors.

### *α*NAC and *γ*-taxilin transfection

HeLa S3 cells were transfected with pCMV plasmid containing the full-length *α*NAC or *γ*-taxilin using Lipofectamine LTX (Invitrogen) or Effectene (Qiagen) transfection reagents according to the manufacturer's protocol.

### Assessment of apoptosis, autophagy, and cell viability

Apoptosis was assessed after incubating cells with Annexin V-FITC (Sigma) or Annexin V-Cy3 (Sigma) at room temperature for 10 min. Cells positive for Annexin were analyzed by FACS scan (Epics XL; Beckmann Coulter, Brea, CA, USA). Cell viability was measured by a modified MTT dye reduction assay using WST-8 (2-(2-methoxy-4-nitrophenyl)-3-(4-nitrophenyl)-5-(2,4-disulfophenyl)-2*H*-tetrazolium, monosodium salt) (Dojindo Laboratories, Kumamoto, Japan). Viable cell fractions were determined as ratios of WST-8 values obtained from treated cells relative to the values of untreated cells. Autophagy was assessed by western blot analysis using LC3-II-specific antibodies and by blocking autophagosome–lysosome fusion using bafilomycin A1 (Sigma).

### Immunofluorescence microscopy

Cells were washed with phosphate-buffered saline (PBS) and fixed with 4% paraformaldehyde in PBS for 20 min and the cells were then permeabilized with 0.2% Triton X-100 in PBS for 15 min at room temperature. Blocking was performed with 1.5% bovine serum albumin and 1.5% skim milk in PBS for 1 h at room temperature. Incubation with the primary antibody was performed overnight at 4 °C. Visualization of nuclei was achieved by incubating the cells with DAPI (1 *μ*g/ml) for 10 min at room temperature. Immunofluorescence visualization was carried out under a TCS SP2 AOBS confocal microscope (Leica Microsystems, Mannheim, Germany).

### Protein extraction, cell fractionation, and western blot analysis

Cells were collected, washed in ice-cold PBS, and lysed in a buffer containing 10 mM HEPES (pH 7.9), 1.5 mM MgCl_2_, 10 mM KCl, 0.5 mM DTT, and a protease inhibitor cocktail (Roche, Basel, Switzerland). For the cytochrome *c* release assay, cells were lysed and fractionated into mitochondrial and cytosolic fractions using a Mitochondria Isolation Kit (Thermo Scientific, Rockford, IL, USA). After centrifugation at 12 000 × *g* for 15 min, the supernatants were pooled as the cytosolic fraction and the pellet for the mitochondrial fraction. The cytosolic fraction was concentrated by using a Microcon centrifugal filter (YM-10; Millipore, Bedford, MA, USA). Equal amounts of proteins were then analyzed on a 15% polyacrylamide gel.

### Mass spectrometry

Total cell lysates were extracted from HeLa S3 cells that were cultured in normoxic or hypoxic conditions for 4–16 h. The protein extracts were digested with trypsin, and the peptides were labeled with iTRAQ reagent and enriched for phosphopeptides using iTRAQ Reagent-Multiple Assay Kit and Titansphere Phos-Tio Kit according to the manufacturer's instruction (Ab Sciex, Tokyo, Japan). Mass spectrometry analysis was performed on the iTRAQ-labeled extracts by using AB SCIE TripleTOF 5600 system and DiNa system (Filgen, Nagoya, Japan). The mass spectrometry spectra were extracted and searched for phosphorylation on Ser, Thy, and Tyr using Protein Pilot Software version 4.5 (Ab Sciex).

### Mouse brain slice culture and immunohistochemistry

Transverse brain slices (500 *μ*m thick) were obtained from newborn (7–8 days) C57BL/6J mice using a tissue chopper (Stoeling, Wood Dale, IL, USA). The slices were then placed on the bottom of Cell Culture Insert (BD Falcon, Franklin Lakes, NJ, USA) in a 6-well dish (Companion Plate; BD Falcon) and were cultivated on the surface of MEM culture medium supplemented with 25% Hank's balanced salt solution and 25% heat-inactivated horse serum (Life Technologies, Carlsbad, CA, USA) under normoxic conditions or hypoxic conditions. The brain slices were fixed in phosphate-buffered 4% paraformaldehyde in PBS (pH 7.4) 48 h after the start of cultivation at room temperature, and embedded in paraffin. Six-micrometer sections were sequentially treated with EDTA-TBS buffer (pH 9.0) at 95 °C for 20 min and 0.3% H_2_O_2_, and then stained with the *γ*-taxilin- or *α*NAC-specific antibodies. The proteins were detected by using Histofine Simple Stain MAX-PO (Nichirei Biosciences, Tokyo, Japan). For western blot analysis, the proteins were extracted using T-PER 78510 Kit (Pierce, Rockford, IL, USA).

### Human brain tissues

Paraffin-embedded brain tissue sections were obtained from different parts (temporal, frontal and occipital lobes, hippocampus, amygdala, and pre- and postcentral gyruses) of the brains of seven patients with AD, or from the corresponding parts of the normal brain (BioChain Institute, Newark, CA, USA).

### Antibodies

The antibodies used in the present study included: *γ*-taxilin (Santa Cruz Biotechnology, Santa Cruz, CA, USA (SC-47462) and Sigma (HPA 000841)), *α*-taxilin (Santa Cruz Biotechnology; SC-33761), *α*NAC (Abnova, Taipei, Taiwan; H00342538), ATF4 (Santa Cruz Biotechnology; SC-200), ATF6 (Imgenex, San Diego, CA, USA; IMG-273), BiP (KDEL; Stressgen, Ann Arbor, MI, USA; SPA-827), cytochrome c (BD Pharmingen; 556433), CHOP (Cell Signaling, Danvers, MA, USA; 2895), PERK (Santa Cruz Biotechnology; SC-13073), phospho-PERK (Santa Cruz Biotechnology; SC-32577), IRE1*α* (Cell Signaling; 3294), phospho-IRE1*α* (Novus, Littleton, CO, USA; NB100-2323), caspase-4 (Stressgen; AAM-114), cleaved caspase-9 (Cell Signaling; 9501, 9505), ubiquitin (FK2; Biomol, Plymouth Meeting, PA, USA; PW 8810), elF2*α* (Cell Signaling; 9722), phospho-elF2*α* (Cell Signaling; 9721), GSK-3*β* (Cell Signaling; 9315), phospho-GSK-3*β* (Cell Signaling; Ser 9: 9336), Bcl-2 (Santa Cruz Biotechnology; SC-7382), Bax (Santa Cruz Biotechnology; SC-7480), Bak (Santa Cruz Biotechnology; SC-832), Bim (BD Pharmingen; 559685), Puma (Cell Signaling; 4976), JNK (Cell Signaling; 9252), phosphor-JNK (Cell Signaling; 4668), XBP-1 (Santa Cruz Biotechnology; SC-7160), Tau (Abcam, Tokyo, Japan; ab 32057, ab 64143), phosphorylated Tau (Abcam (pT231: ab 151559, pS404: ab 92676); Invitrogen; pS396: 44752G), NFT (Dako, Glostrup, Denmark (40024) and Invitrogen (AHB0161)), LC3A/B (Cell Signaling; 12741), SQSTM1/p62 (Cell Signaling; 8025), and *β*-actin (Santa Cruz Biotechnology (SC-1615) and Sigma (A3853)).

## Abbreviations

*α*NAC: nascent polypeptide-associated complex α-subunit
GSK-3*β*: glycogen synthase kinase 3*β*
UPR: unfolded protein response
ER: endoplasmic reticulum
HIF: hypoxia-inducible factor
mTOR: mammalian target of rapamycin
IRE: inositol-requiring protein
PERK: protein kinase RNA-like endoplasmic reticulum kinase
ATF: activating transcription factor
BiP: intraluminal chaperone Ig-binding protein
NC: nascent chain
eIF: eukaryotic initiation factor
CHOP: C/EBP homologous protein
XBP-1: X-box-binding protein
JNK: c-Jun N-terminal kinase
Bcl-2: B-cell lymphoma 2
Puma: p53-upregulated modulator of apoptosis
NFT: neurofibrillary tangle
AD: Alzheimer's disease
Bak: Bcl-2 homologous antagonist/killer
Bax: Bcl-2-associated X protein
BH3: Bcl-2 homology domain
Bim: Bcl-2-interacting mediator of cell death
