# Supplementary figures and images for: GSK-3β-dependent downregulation of γ-taxilin and αNAC merge to regulate ER stress responses
Source: Cell Death Dis. 2015 Apr 16;6(4):e1719–. doi: 10.1038/cddis.2015.90 (PMC4650556; doi:10.1038/cddis.2015.90)

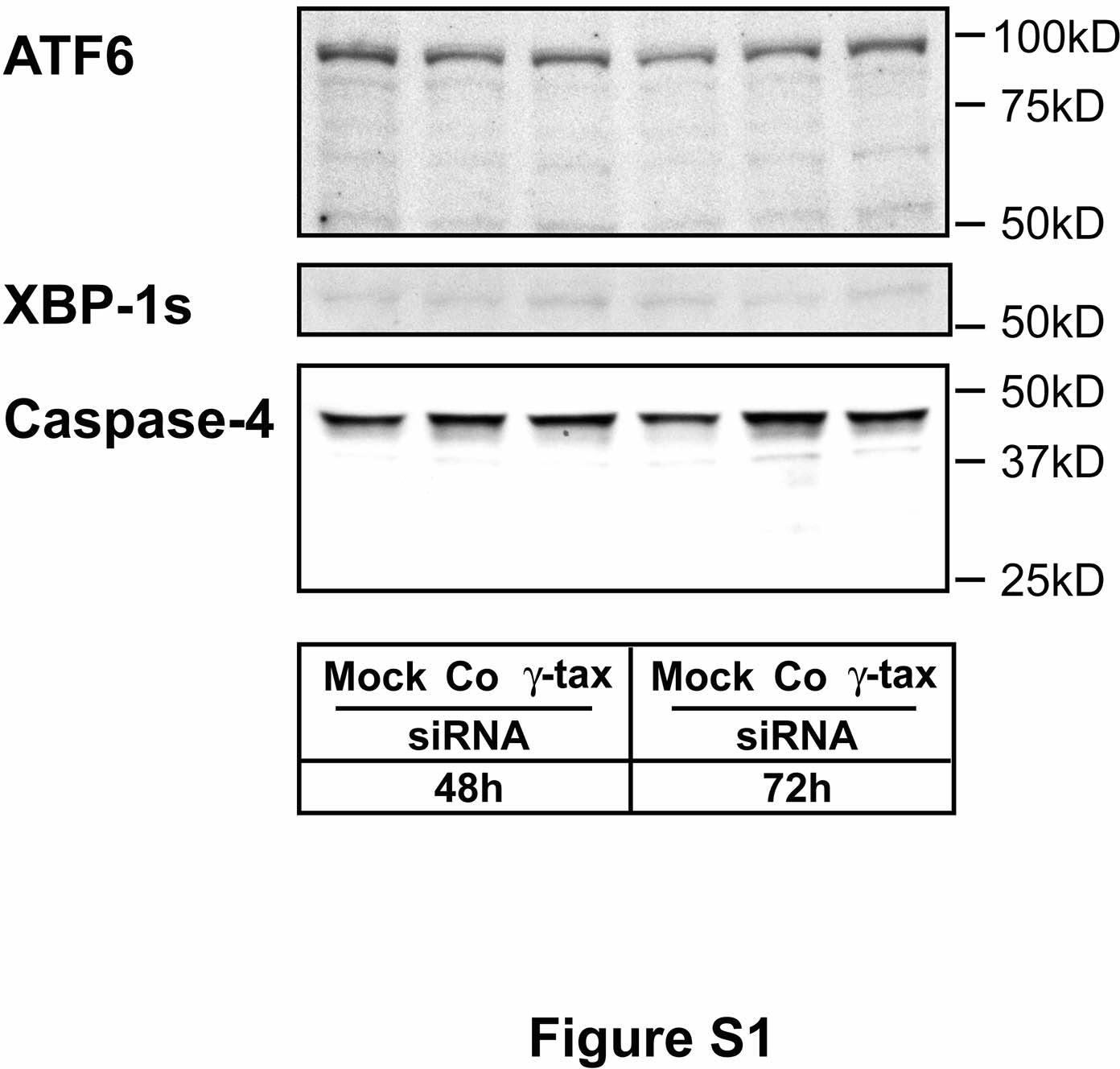

Supplement: Supplementary Figure 1 [file cddis201590x1.tif]

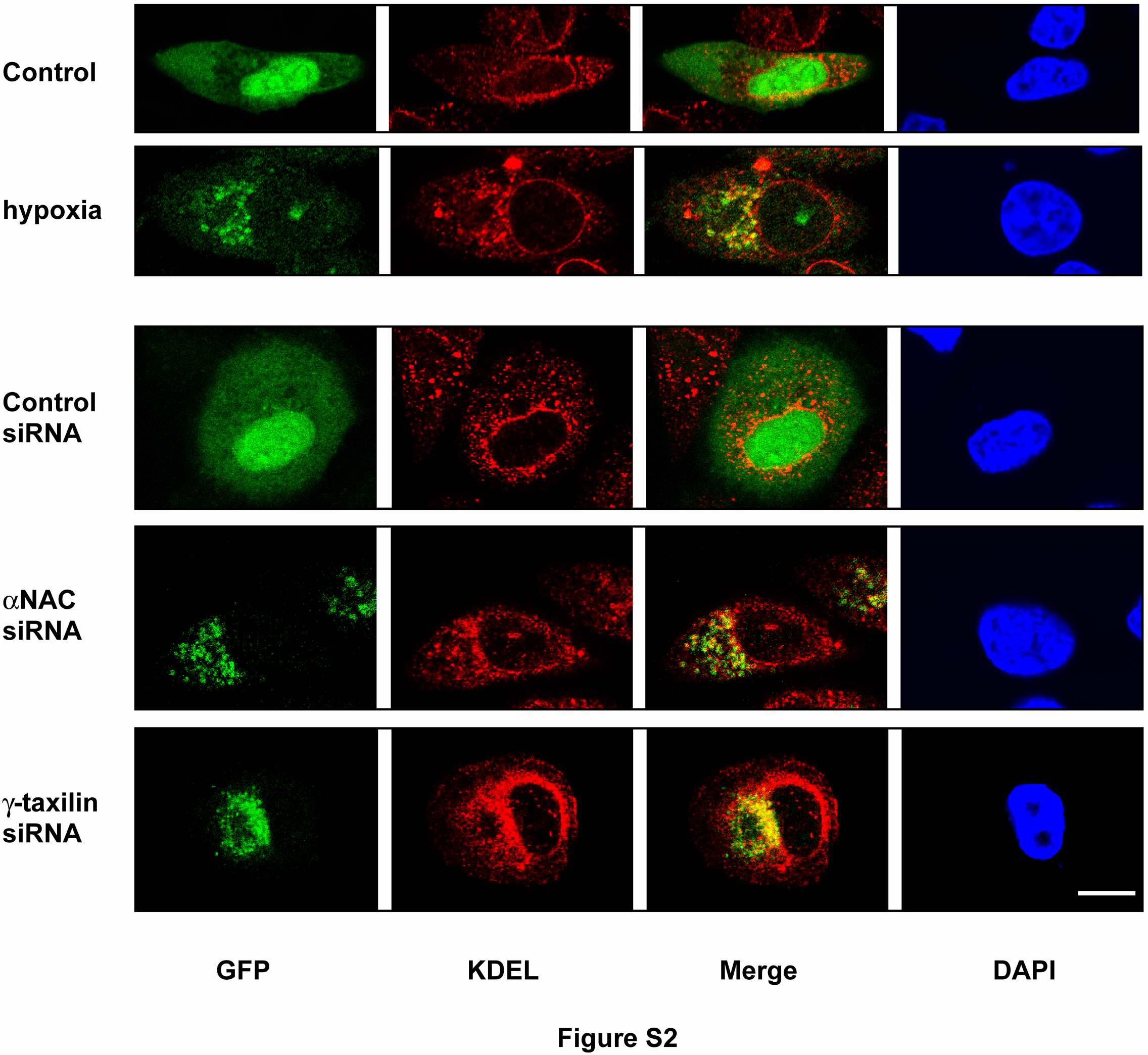

Supplement: Supplementary Figure 2 [file cddis201590x2.tif]

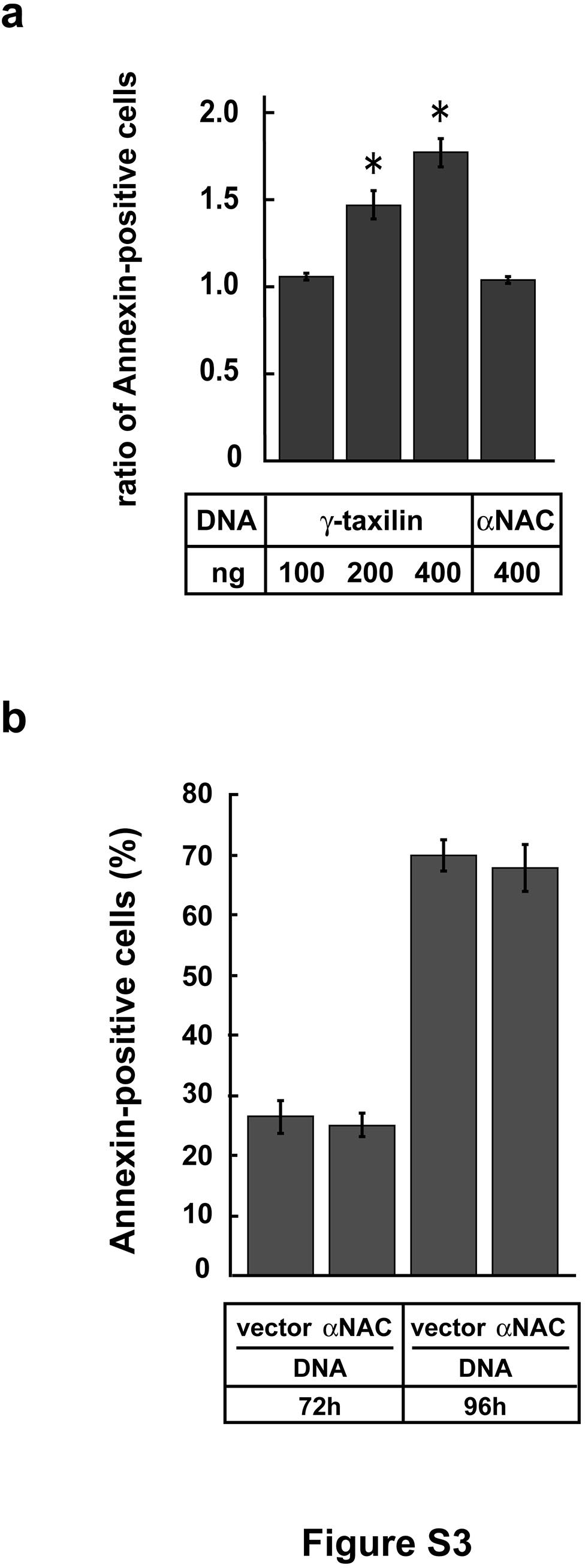

Supplement: Supplementary Figure 3 [file cddis201590x3.tif]

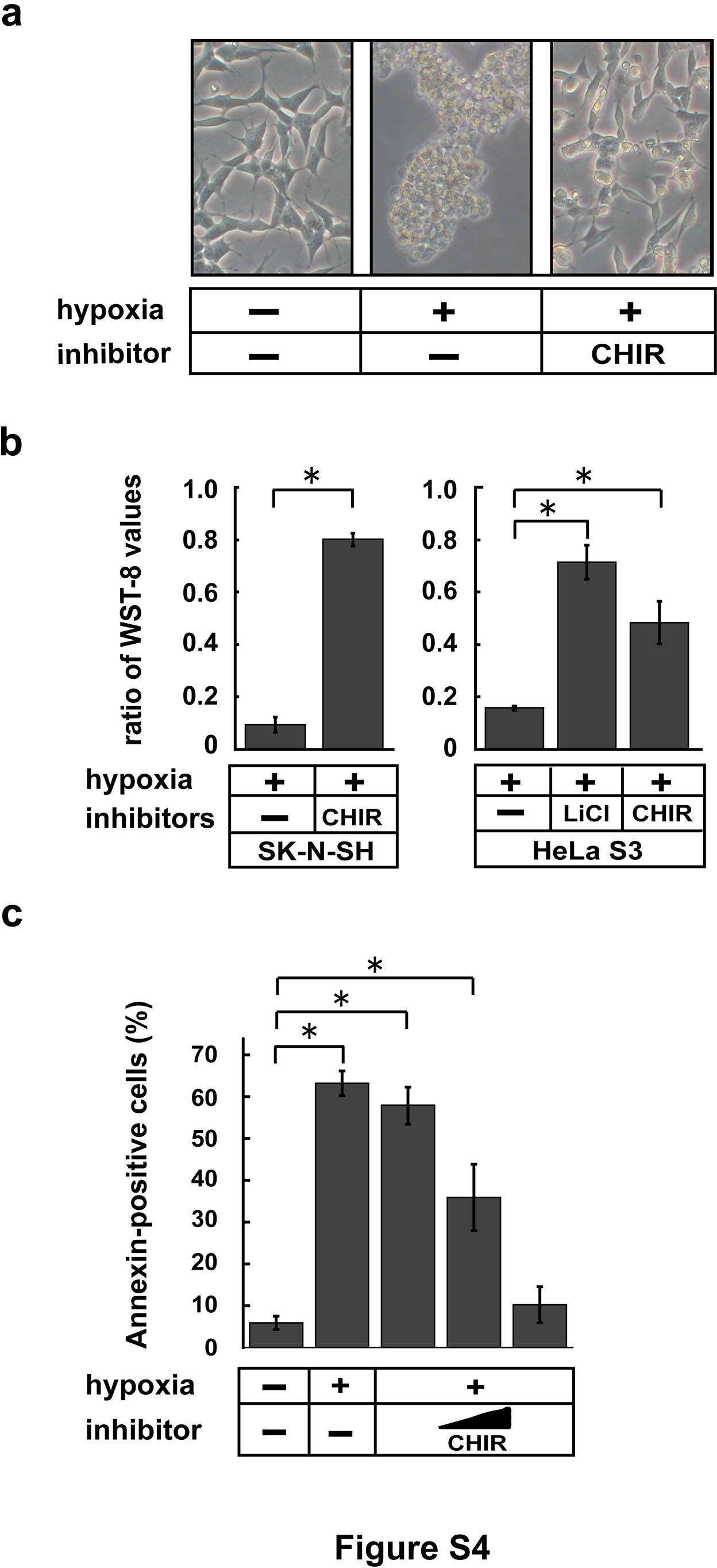

Supplement: Supplementary Figure 4 [file cddis201590x4.tif]

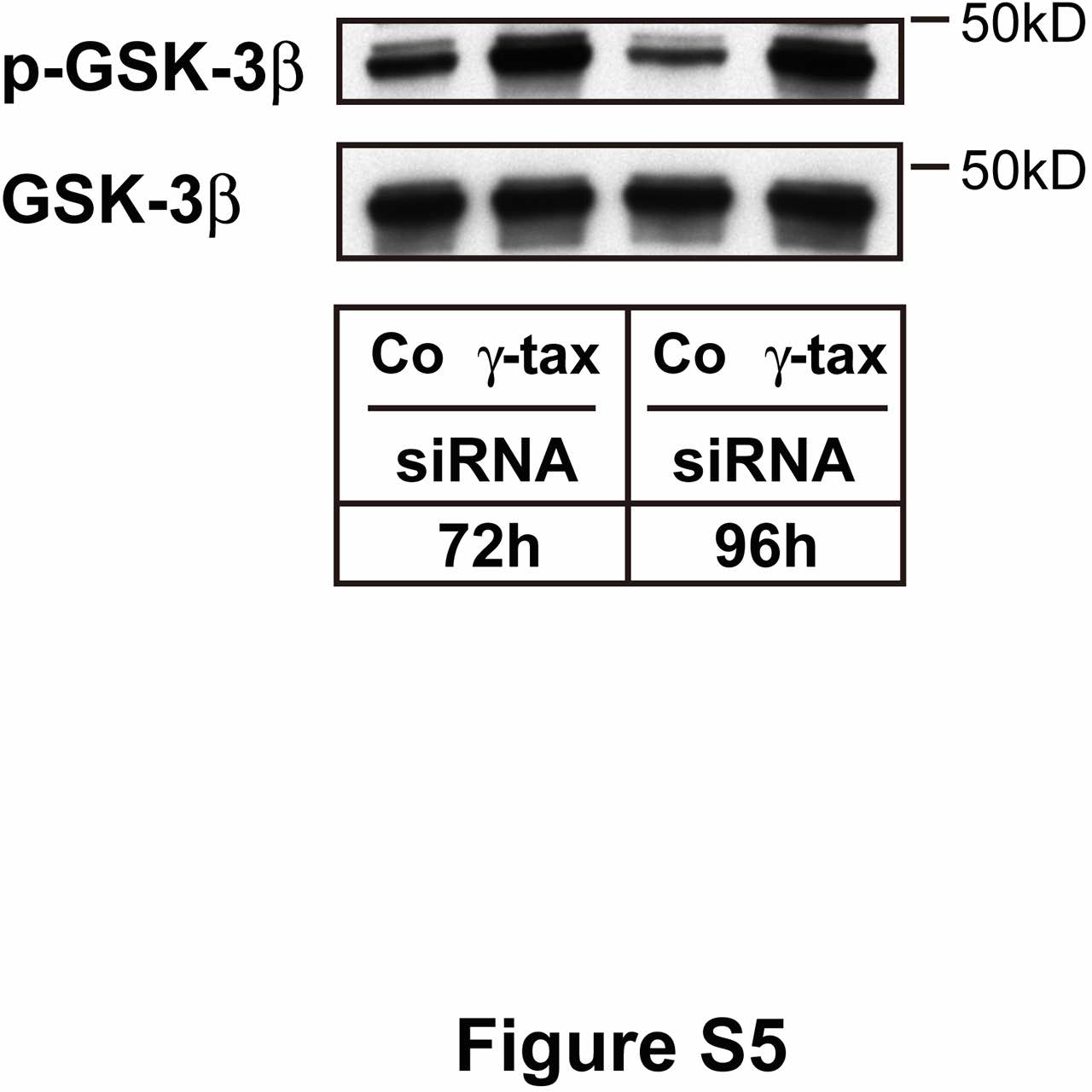

Supplement: Supplementary Figure 5 [file cddis201590x5.tif]
