# Supplementary material for: GSK-3β-dependent downregulation of γ-taxilin and αNAC merge to regulate ER stress responses
Source: Cell Death Dis. 2015 Apr 16;6(4):e1719–. doi: 10.1038/cddis.2015.90 (PMC4650556; doi:10.1038/cddis.2015.90)
Supplement: Supplementary Figure Legends [file cddis201590x6.doc]

**Fig. S1.—**γ**-taxilin ablation does not activate ATF6, XBP-1, or caspase 4.**

HeLa S3 cells were treated with solvent alone (Mock), control siRNA (Co), or γ-taxilin siRNA (γ-tax) for 48 h and 72 h and the expression levels of ATF6, spliced XBP-1, and caspase 4 were determined by Western blotting.

**Fig. S2.—Altered intracellular localization of exogeneously expressed GFP in γ-taxilin or αNAC siRNA-treated HeLa S3 cells.**

Confocal microscopy shows intracellular accumulations of exogeneously expressed GFP (green) in control, hypoxic, control siRNA-treated, γ-taxilin siRNA-treated, or αNAC siRNA-treated HeLa S3 cells. At this stage, γ-taxilin and αNAC siRNA-treated cells do not exhibit apoptotic nuclei (DAPI). KDEL-positive cytoplasmic structures indicate ER. Scale bar, 10 μm.

**Fig. S3.—Effects of overexpressing** γ-**taxilin or** α**NAC in normoxic and** γ-**taxilin siRNA-treated cells.**

(**a**) γ-taxilin or αNAC was transiently transfected in HeLa S3 cells. Percentages of annexin-positive cells were determined 48 h after the transfection. Bar graph shows percentage ratios of annexin-positive cells that were transfected with γ-taxilin or αNAC relative to those of annexin-positive cells that were transfected with vector alone (p <0.001, t- test). Bar graph shows means ± s.d. (n = 3). *Significant differences in percentages between cells treated with γ-taxilin- or αNAC and those treated with vector alone. (**b**) HeLa S3 cells were treated with γ-taxilin siRNA for 48 h and then the cells were transfected with αNAC or vector DNA for indicated times. Bar graph shows means ± s.d. (n = 3).

**Fig. S4**.**—Pharmacological inhibition of GSK-3β suppresses hypoxia-induced cell death.**

(**a**) Phase-contrast micrograph shows that CHIR suppresses cell floating in hypoxic conditions. (**b**) LiCl, or CHIR 99021 increased cell viability of hypoxic SK-N-SH and HeLa S3 cells. (**c**) Annexin assay shows a dose-dependent (3, 10, and 15 μM of CHIR 99021) suppression of apoptosis in hypoxic SK-N-SH cells. (**b, c**) Data are shown as means ± s.d. (n = 3). *Significant differences (p <0.001, Tukey-Kramer test or t-test).

**Fig. S5.-- γ-taxilin ablation inhibits GSK-3β activity.**

γ-taxilin RNA interference (72 and 96 h after addition of siRNA in the culture medium) upregulates phosphorylated GSK-3β in SH-SY5Y cells.
